# A Measurement Equivalence Study of the Family Bondedness Scale: Measurement Equivalence Between Cat and Dog Owners

**DOI:** 10.3389/fvets.2021.812922

**Published:** 2022-01-11

**Authors:** William R. Nugent, Linda Daugherty

**Affiliations:** ^1^College of Social Work, The University of Tennessee, Knoxville, Knoxville, TN, United States; ^2^Social Work Office of Research and Public Service, College of Social Work, The University of Tennessee, Knoxville, Knoxville, TN, United States

**Keywords:** human-animal bond, measurement of HAB, comparing attachment to cats and dogs, measurement equivalence of HAB measures, family bondedness scale

## Abstract

About 38.4% of U.S. households include a dog, and 25.4% a cat, as pets, and a recent poll suggested over 90% of pet owners feel their companion animal is a family member. Numerous studies have suggested pet ownership has physical, mental, and social health benefits, though much of this research has yielded mixed results. Results of a recent review suggested significant measurement problems in human-animal interaction (HAI) and human-animal bond (HAB) research, including the absence of validity evidence, overly long measures, lack of evidence for measurement equivalence across species of pets, and measures lacking a basis in important psychological, family, and attachment theories. This article describes the development and results of a measurement equivalence study of a new measure of the HAB called the family bondedness scale (FBS). This scale, and the research results, address multiple gaps in HAB measurement. Results of multi-group confirmatory factor analyses with multiple covariates indicated the scores on the FBS showed equivalence between cat and dog owners. The use of the FBS in both veterinary research and practice, as well as in research and practice in other disciplines, such as social work and psychology, are considered.

## Introduction

According to (1) American Veterinary Medical Association statistics (https://www.avma.org/resources-tools/reports-statistics/us-pet-ownership-statistics), about 38.4% of U.S. households include a dog, and 25.4% a cat, as companion animals. About 2.8% include birds, and fewer include such exotic companion animals as fish, snakes, rabbits, and other animals. The 2017-2018 American Pet Products Association (APPA) survey (https://www.mceldrewyoung.com/wp-content/uploads/2018/08/2017-2018-Pet-Survey.pdf) found 68% of U.S. households included a pet: 48% one or more dogs, 38% a cat, 10% fresh water fish, 6% a bird, 4% reptiles, 2% small animals, and 2% a horse. A recent Harris poll found 95% of U.S. respondents felt their pets to be members of their family (2), for example, dogs are often referred to as “fur babies” by owners who see themselves as their dog's “parents” (3). These numbers hint at the importance of human-animal interaction (HAI) and the human-animal bond (HAB) for persons and families in the U.S.

There is evidence of potential benefits of animals, the so-called “pet effect,” for physical, mental, and social health. Talking to and/or petting a companion animal has been found to lower blood pressure (4), even when the companion animal is a snake (5). Research has found children exposed to pets early in life tend to have lower levels of asthma and allergic rhinitis (6, 7), increased abundance of bacteria negatively associated with childhood atopy and obesity (8), and Kates et al. (9) found pets may influence gut microbiota so as to reduce atopic diseases. Pet ownership may be associated with reduced loneliness, anxiety, depression, and increased exercise (10), associated with such health benefits as lower blood pressure (11–13), and may be associated with longer survival after discharge from a coronary care unit and decreased heart attack mortality (14, 15). A recent critical literature review and meta-analysis of 10 studies between 1950 and 2019, involving 3,837,005 persons, found dog ownership associated with a 24% risk reduction for all-cause mortality as compared with non-ownership (13). A second recent meta-analysis (16) of 12 studies, involving 488,988 persons, found in subgroup analyses pet ownership was associated with lower cardiovascular disease mortality in the general population, and found pet ownership associated with lower adjusted cardiovascular disease risk in patients with established cardiovascular disease. There may be other benefits associated with pet ownership and the human-animal bond (17, 18).

The research in many of these areas of health and mental health has, however, been mixed and inconsistent and further research is needed to resolve the varied results (19–21). For example, a study of 425 heart-attack victims found pet owners were more likely than non–pet owners to die or suffer remissions within a year of a heart attack [22 vs. 14%; (22)]. Other research found that doing a stressful task in the presence of a dog had no short-term effect on blood pressure (23). The problems of inconsistent results led Herzog (19) to argue that (p. 236), “… the existence of a generalized “pet effect” on human mental and physical health is at present not a fact but an unsubstantiated hypothesis.” Herzog (19) urged further research on the effects of companion animals with greater methodological rigor.

As the definition of family has evolved in the United States, some pet owners now consider themselves as “pet parents” (24–26). There has been recent interest in pet parenting and the effects of different parenting styles on the relationships and bonds pet owners have with their pets, as well as on pet behavior and health. There has been speculation that the ways in which pet owners interact with their pets can influence the bond they have with their companion animals (27, 28), though few studies have investigated this hypothesis. The relationship between parenting and human-dog interaction styles and canine obesity has been studied, and the relationship between parenting styles and the way dogs respond to the threatening approach of a stranger has been investigated (29, 30). It has also been inferred that owner-dog interaction and human caregiving styles may have implications for avoiding undesired dog behaviors associated with relinquishing a canine pet (31). Thus, pet parenting and interaction styles may influence the bond owners have with their pet, and vice versa, the bond may influence owner behavior toward their companion animal. This is another area of needed research.

Rodriguez et al. (32) reviewed measures used in HAI and HAB research. They argued measurement problems have significantly hindered HAI and HAB research, a view echoed by Dwyer et al. (33). Rodriguez et al. (32) identified the scarcity of measures with strong evidence of validity for measuring important constructs in HAI and HAB research; the lack of evidence for reliability; the lack of brief measures; and the lack of measures with grounding in attachment, family, and psychological theories as problems. They noted a recent study (34) identified numerous measures for use in HAI research, including measures of attitudes toward animals, attachment to animals, and bonding measures. However, they concluded there was a critical scarcity of validity research on the scores from these measures. Many of these measures are long and the need for short, rapid assessment measures of the HAB has been underscored (35). Dwyer et al. (33) recommended psychometric research was needed on measures of the HAB that included reliability estimates, validity studies, studies demonstrating adequate factor loadings on the latent construct represented by scores on the measure, and measurement equivalence studies focusing on among other forms of equivalence, equivalence across different species.

As Wilson and Netting (34) found, numerous measures of the HAB exist, and Anderson (36) gathered a number of these measures into a book. Several have been developed for use with children, such as the CENSHARE Pet Attachment Scale (37), the Companion Animal Bonding Scale [CABS; (38)], and the Pet Attachment Scale [PAS; (39)]. The Pet Bonding Scale [PBS; (40)] was developed for use with pre-adolescents. The Lexington Attachment to Pets Scale (LAPS) was developed using items from pet attitude scales and from the CABS and was intended for use with both owners of dogs and owners of cats (41). More recently the Monash Dog Owner Relationship Scale (MDORS) was developed for use in measuring facets of owner's relationships with their dogs (33). Howell et al. (42) used the MDORS as a base for development of the Cat-Owner Relationship Scale (CORS).

Perhaps the most commonly used measure of the human-animal bond in research has been the Lexington Attachment to Pets Scale [LAPS; (41, 43)]. This scale has been used in a number studies on the HAB [see (36), for a partial listing]. Zaparanick (43) conducted a psychometric study of the LAPS, with findings that challenged the validity of scores from this scale. While most items appeared to represent some aspect of an emotional bond, content validity challenges were raised about some items. For example, content validity issues can be raised about items c, “I believe that pets should have the same rights and privileges as family members,” and item n, “Pets deserve as much respect as humans do,” both of which appear to measure beliefs about animal welfare or animal rights as opposed to the HAB. The LAPS also includes reverse-scored items, a structural aspect shown to introduce scoring factors and adversely affect validity (44). Zaparanick's (43) results also raised questions about the factor structure of the LAPS. She argued that scores on the LAPS were not equivalent in the sense needed for valid comparisons of bondedness to animals of different species. Among other recommendations, she suggested placing the name of a pet/companion animal in items might help increase validity.

The MDORS is a multidimensional scale, based on social exchange theory, with three sections, or subscales: a factual section (example item: How often do you groom your dog?), a second component that the authors view as reflecting “perceived emotional closeness” (example item: How traumatic do you think it will be when your dog dies?), and a third section “perceived costs” (example item: My dog makes too much mess). The second of these sections contains items that on the face of it represent the degree to which a respondent feels an emotional bond with their dog, and the reliability estimate for scores on this section of the MDORS was 0.84 in Dwyer et al. (33) study. One limitation in this subscale is that it contains multiple Likert type scoring rubrics. One scoring metric is a 5-point agree/disagree scaling for items such as, “My dog helps me get through the tough times.” A second is a 5-point frequency of occurrence scaling for items such as, “How often do you tell your dog things you don't tell anyone else?” The third is a 5-point degree of trauma scaling for items such as, “How traumatic do you think it will be for you when your dog dies?” Here is how this is a problem. Imagine the response to an item on the agree/disagree scaling is four, and a response to an item on the frequency of occurrence scaling is four, and the response to an item on the degree of trauma scaling is four, giving a sum over these items of 12. Interpreting this overall score as representing the level of magnitude of a single latent construct is logically problematic. The three different scaling metrics do not appear to be conceptually equivalent. The assumption that a score of four means the same thing in terms of agreement, frequency of occurrence, and degree of trauma makes no conceptual sense. There could therefore be construct irrelevant variance introduced into the total scale scores over the items, a threat to construct validity (45). The MDORS was created for use with dog owners.

The CORS was developed for use with cats (42). If the MDORS and CORS were to be used in an effort to compare the HAB between dog and cat owners, an equating study would need to be done in order to enable direct comparison of scores (46). This limits the use of these scales in studies comparing the HAB between cat and dog owners.

Branson et al. (47) noted the LAPS and other measures of the HAB do not producing scores valid for specifically comparing bonded levels between dog and cat owners. Zasloff (48) made this same argument but in general for comparing HAB levels across different animal species. Zasloff pointed out that wording matters in these measures, as the inclusion of the species of the pet (cat or dog) in items influenced the scores from the measures. This issue is a lack of measurement equivalence between responses from dog and cat owners specifically, and between owners of different species of pets in general. Measurement equivalence concerns the extent to which a scale or measure works the same for different groups (49). Pendergrast et al. (50) emphasized the critical role of measurement equivalence studies as part of instrument development and the essential role of measurement equivalence as a form of validity evidence. As far as the authors of the current study have been able to determine, no studies have investigated the measurement equivalence of scores from measures of the HAB for scores from dog and cat owners or for comparing scores from owners of any different species. This deficiency makes it more difficult to do research in which the HAB is compared between dog and cat owners, or between owners of pets of any different species (49, 50).

These limitations of extant measures of the HAB, along with the growing evidence pet owners feel their companion animal is a family member, stimulated the development of a new scale to measure the HAB. The current study focused on (1) the creation of a scale, the Family Bondedness Scale, measuring the degree to which a person feels emotionally bonded to a companion animal as an integral part of their family, a concept referred to as “family bondedness,” defined below, and (2) investigation of the measurement equivalence of the scores from the scale for responses from persons concerning their family bondedness to cats and dogs.

## Scale Creation

Family bondedness is defined as the condition in which a person feels a positive valence emotional bond to a pet in a manner approaching, if not equivalent to, their positive valence emotional bond to a human family member. This positive emotional bond is characterized by love and affection and an emotional sense the pet is a member of their immediate family. The Family Bondedness Scale (FBS) was designed to be used with adults 18-years-old and older. It was designed to be a unidimensional scale the scores from which represent the degree to which a person is emotionally and affectionately bonded to a pet as a member of their family. It was designed to be a rapid assessment scale (51) suitable for use in HAI and HAB research by veterinarians, psychologists, social workers, and others, and simultaneously convenient for use by professionals in a wide range of fields for assessing the degree to which persons feel emotionally bonded to a companion animal in a manner equivalent with their emotional bond to human family members. It was also intended to be applicable in studies comparing the family bondedness of pet owners who own pets of different animal species.

Content validity was emphasized from the beginning of development of the FBS. Existing scales for measuring the human-animal bond were reviewed for examples of item content, in particular the LAPS, CABS, and PBS scales. These reviews were used to generate potential item content. While no items reviewed were used verbatim on the FBS, some FBS items had wording similar to that on other scales. For example, item two on the FBS reads, “I feel [pet's name] is a member of my family,” while item t on the LAPS reads, “I feel that my pet is a part of my family.” A focus group was conducted in which experienced veterinarians, veterinary technicians, and other employees of veterinary clinics were asked what kinds of indicators they observed that, in their experience, suggested persons were emotionally bonded with their pets in a manner commensurate with the pets being family members. The results of this focus group also led to the generation of possible item content.

Following recommendations by Zaparanick (43), the scale was designed so that the names of pets were included in each scale item. Use of the pet's name in items theoretically would evoke the emotional bond persons have with the pet (52). Following Zasloff (48), all items were species non-specific, that is, there no words such as “dog” or “cat” in the items. These two characteristics of items were believed to help insure the construct measured by the items was the degree of emotional bonding in a manner commensurate with their emotional bondedness to human family members. No reverse scored items were created in order to avoid construct irrelevant factors (44). These items were reviewed for good item quality by a Ph.D.-level psychometrician. Based on their recommendations, the 42-items were revised to meet criteria for psychometrically sound items.

A total of 43 items were created, and then the 42 items were reduced in number to 23 by removing items that appeared to be duplicative in terms of specific content. The result was a Likert-type scale with 23 items, a number consistent with the numbers of items on rapid assessment instruments (51), scored on a 5-point agree/disagree category partition. The scaling was such that higher scores were indicative of higher family bondedness with a companion animal, and vice versa, with possible scores ranging from 23 to 115. This wide range of possible scores was designed to ensure the possibility of a wide range of scores in research. A wide range of scores would help increase the reliability of scores and reduce the possibility that restriction of range of scores would inhibit the ability to detect correlations between scores on the FBS scale and other variables of interest (53).

**Figure 1:**
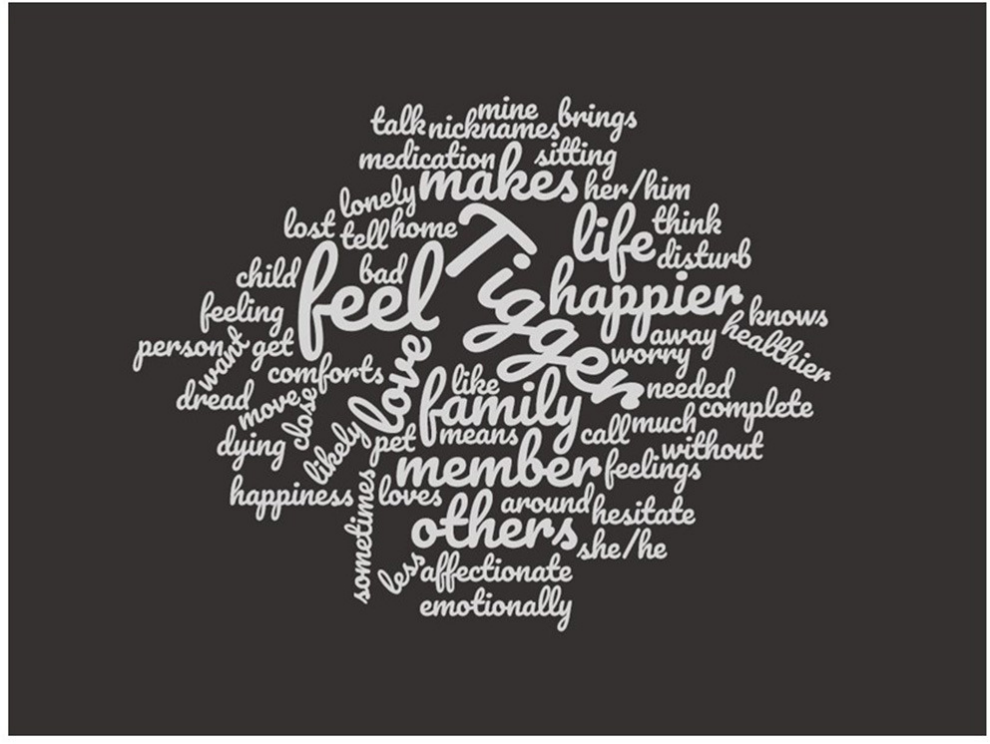
Word cloud of words in items on the Family Bondedness Scale. Larger font words are those appearing more frequently in Family Bondedness Scale items, and vice versa. It was assumed in the creation of this cloud that the name of the pet was “Tigger,” so this word is largest in the word cloud.

Table 1 shows the items on the FBS, while Figure 1 shows a word cloud of the words in the items on the FBS assuming the pet's name is “Tigger.” The larger words in the cloud are the most frequently occurring, and vice versa. This visualization is a form of evidence of content validity (54).

**Table 1:** Bonded family scale items.

I love [pet's name].
I feel [pet's name] is a member of my family.
I feel [pet's name] is like a child of mine.
I sometimes hesitate to move when sitting
by [pet's name] because I do not want to disturb her/him.
I would feel lost witdout [pet's name].
[Pet's name] brings happiness to my life.
I dread [pet's name] dying.
I talk to [pet's name] as if she/he is a person.
I tdink [pet's name] knows what I am feeling.
[Pet's name] being in my family makes me happier.
[Pet's name] makes my family feel more complete.
Having [pet's name] in my life makes me feel less lonely.
I call [pet's name] by affectionate nicknames.
I love to pet [pet's name].
When I am away from home I worry about [pet's name].
[Pet's name] comforts me when I have bad feelings.
I tell otders tdat [pet's name] is a member of my family.
Being witd [pet's name] makes me happier.
[Pet's name] means as much to me as otders in my family.
I feel emotionally close to [pet's name].
I feel [pet's name] loves me.
I am more likely to get needed medication for [pet's name] tdan for myself.
I feel having [pet's name] around makes me healtdier.

### Readability

The readability of the final 23-item scale was assessed following methods used by Paasche et al. (55) in their assessment of readability of informed consent documents. Ten online readability calculators were used to assess the readability of the scale and its items. The Flesch-Kincaid grade level index was used as the readability index. The estimated mean Flesch-Kincaid grade level score was 3.4, t_(9)_ = 6.8, *p* < 0.05, 95% CI (2.3–4.6). These results were consistent with the easy readability of the scale and its items.

## Methodology

### Human Subjects

Researchers obtained IRB approval for the current study from the University of Tennessee IRB on April 20, 2020. The study number assigned was UTK IRB-20-05773-XM.

### Sampling

The objective of the purposive sampling plan was to obtain a cross-sectional national sample. Quotas were established to ensure responses from a stratified sample based upon gender and age of the pet owner and by type of pet. Three age strata were created for male and female dog and cat owners. The sample goal was a minimum of 200 responses on the family bondedness scale from each of the following groups: female cat owners, male cat owners, female dog owners, and male dog owners. This would give an overall sample size of at least 800, enabling analyses testing measurement equivalence across these different groups with a minimum of 200 per group (56). The study was completed utilizing a web panel provided by the market firm Dynata.

### Measurement

Respondents were asked to complete the family bondedness scale with respect to their bond to the pet they had owned the longest. The name of this pet/companion animal was inserted into each item on the FBS scale, for example, the first item on the scale would read, “I love “pet's name,”” or as an illustration, “I love Tigger.” The logic for this methodology was that including a companion animal's name in the item stem would more strongly arouse the respondent's emotional bondedness to the animal than the more affectively neutral word “pet.” Respondents were also asked to give demographic information on their gender identity, type of housing they lived in, type of setting in which they lived (rural, suburban, or urban), ethnicity (Hispanic or non-Hispanic), self-identified race, education level, and marital status. These variables were to be used as covariates in a latent variable regression that was a part of the Multiple-Group Confirmatory Factor Analysis/Multiple Indicators Multiple Causes (MGCFA-MIMIC) analysis, as described below. The survey was created using Qualtrics.

### Research Design

The study employed a cross-sectional national web survey.

#### Data Analyses

Multi-Group Confirmatory Factor Analysis with Covariates, or MGCFA-MIMIC, analysis methods were used as described by Brown (56) using Mplus version 7. This involved fitting a multiple group Confirmatory Factor Analysis (CFA) model, with a regression of the family bondedness latent construct on the following independent variables: self-identified gender identity, region where respondents lived (rural, suburban, urban), self-identified race, marital status, education level, income, type of housing respondents lived in, and ethnicity (Hispanic, non-Hispanic). This analysis was done using weighted least squares mean-variance adjusted (WLSMV) estimation, which is appropriate for ordinally scored items such as on the FBS, and the Theta parameterization was employed (50). This method provided a test of measurement equivalence between FBS scores for family bondedness between cats and dogs controlling for the relationships between the independent variables and the latent construct (56). The suggestion of statistically significant paths from any of the independent variables to FBS items would indicate, in this analysis, the existence of lack of measurement equivalence for the item as a function of the independent variable, a condition referred to as differential item functioning, or DIF. The possibility of these paths would be indicated by statistically significant modification indices (56). The measurement hypothesis was the FBS is a unidimensional scale. Consistent with the multi-evidence approach to measurement equivalence recommended by Pendergast et al. (50), reliability estimation, using Chronbach's coefficient alpha, and corrected item-total correlations using SPSS version 27 that focused on measurement equivalence were also conducted.

## Results

### Characteristics of Sample

There was a total of 836 responses to the survey that were obtained from persons in 49 states. In response to a query about respondent's gender identity, 51.3% identified themselves as female, 46.7% as male, 1.0% as non-binary, 0.5% as third gender, 0.2% preferred to not describe their gender identity, and 0.4% preferred to not respond to this question. Fifty-four-point-eight percent of respondents were married, 6.9% were living with a partner, 9.0% were divorced, 1.3% were separated, 2.5% were widowed, 25% were single, and 0.5% refused to answer the question about marital status. Thirty-two-point-eight percent had a bachelor's degree, 22.5% a graduate degree, 26.1% had 1–3 years of college, 15.7% had completed high school or had a GED, 2.3% had 11-years or less of education, and 0.7% refused to answer the query about education.

The mean number of dogs and cats owned by respondents was 2.03 (SD = 2.0), with a range from 1 to 31. A test of normality of this distribution showed it non-normal and highly right-skewed, with 94% owning four or less cats and dogs. A length of pet ownership item revealed 47.8% of respondents had owned a cat the longest, while 52.2% reported they had owned a dog the longest.

Tables 2, 3 show sample percentages of respondent's income brackets, and racial and ethnic breakdowns, respectively, as well as comparisons with U.S. population values. There were no missing data on income or race and ethnicity, though 0.8% responded they were not sure about income, and 3.0% refused to answer this question, and 1.3% were not sure about their Hispanic origin, and 1.1% refused to answer this ethnicity question. Statistical tests showed the sample of pet owners differed with respect to income and racial/ethnic make-up as compared with U.S. population figures as Indicated by the asterisks. Notably, the sample was comprised of a greater percentage of White respondents, a lower percentage of Black/African-American respondents, and a smaller percentage of Hispanic respondents relative to U.S. population percentages. There were also statistical differences in percentages of respondents in the lowest and highest income brackets relative to percentages in the U.S. population. The implications of these differences are considered later.

**Table 2:** Comparisons of sample and U.S. income distributions.

**Income range**	**Sample percentages**	U.S. percentages^*^
< $15,000	6.8	9^**^
$15,000–$29,999	10.2	12
$30,000–$44,999	11.1	12
$45,000–$59,999	10.6	11
$60,000–$74,999	10.8	9
$75,000–$99,999	16.1	12^**^
$100,000–$149,999	17.3	16^**^
$150,000 and greater	13.2	19^**^

* *Percentages from https://dqydj.com/average-median-top-household-income-percentiles/*.

** *Differences between column percentages statistically significant*.

**Table 3:** Comparisons of sample and U.S. racial percentages.

**Race**	**Sample percentages**	U.S. percentages^*^
White	84.3^**^	76.3^**^
Black/African-American	8.5^**^	13.4^**^
Native American	1.8	1.3
Asian	5.3	5.9
Native Hawaiian	0.8^**^	0.2^**^
Mixed	0.6^**^	2.8^**^
Hispanic	12.8^**^	18.5^**^

* *Percentages from https://www.census.gov/quickfacts/fact/table/US/PST045219*.

** *Differences between column percentages statistically significant*.

Table 4 shows a comparison of the educational attainment in the study sample and that in the U.S. according to 2020 census data (https://www.census.gov/data/tables/2020/demo/educational-attainment/cps-detailed-tables.html). This comparison suggested the educational attainment level in the study sample was higher than that in the general U.S. population in that about 84.3% of the study sample had an educational attainment greater than high school level as compared with about 54.6% of the general U.S. population.

**Table 4:** Educational attainment of study respondents as compared with 2020 U.S. Census values^*^.

**Level of education attainment**	**U.S. according to 2020 Census data**	**Current study data**
High school	45.4%	15.7%
Bachelor's degree	36.1%	32.8%
Graduate/Professional degree	18.4%	22.5%

* *Census values from https://www.census.gov/data/tables/2020/demo/educational-attainment/cps-detailed-tables.html*.

There was no relationship between the pet (cat or dog) respondents reported their bond with and gender identity, $\chi_{\left(5\right)}^{2}$ = 4.8, *p* > 0.05; with where respondents lived (rural, suburban, urban), $\chi_{\left(4\right)}^{2}$ = 4.5, *p* > 0.05; or with type of housing, $\chi_{\left(6\right)}^{2}$ = 10.99, *p* > 0.05. There was no relationship between marital status and whether the respondent reported their bondedness with a cat or a dog, $\chi_{\left(6\right)}^{2}$ = 8.6, *p* > 0.05; and no relationship between income and whether the respondent reported their bondedness with a cat or a dog, $\chi_{\left(9\right)}^{2}$ = 15.6, *p* > 0.05.

### Cat or Dog?

Of the 836 respondents, 400 responded to the FBS concerning their bondedness with a cat, while 436 responded with respect to their bondedness with a dog. The mean bondedness score for cat owners was 94.5 (SD = 17.33) and for dog owners 97.3 (15.9). The difference between FBS scores for cat and dog owners was, t_(663)_ = −2.24, *p* < 0.05. While these results suggested dog owners were slightly more bonded to their companion animal than cat owners, this statistically significant difference represented an extremely small effect size, Cohen's d = −0.18 (95% CI−0.33,−0.02), accounting for only about 0.5% of the total variation in bondedness scores. The mean item scores and standard deviations are shown in Table 5.

**Table 5:** Item score means and standard deviations (SD).

**Item**	**Mean score cat**	**Mean score dog**	**SD cat**	**SD dog**
I1	4.52	4.64	0.79	0.66
I2	4.46	4.52	0.83	0.77
I3	3.93	4.10	1.21	1.07
I4	3.99	3.85	1.13	1.11
I5	4.01	4.19	1.12	1.02
I6	4.45	4.52	0.78	0.74
I7	4.32	4.36	0.96	0.92
I8	4.17	4.15	0.95	1.01
I9	3.80	4.09	1.07	0.96
I10	4.36	4.38	0.83	0.84
I11	4.19	4.34	0.94	0.88
I12	4.14	4.26	0.98	0.92
I13	3.89	3.92	1.18	1.22
I14	4.42	4.43	0.79	0.85
I15	3.77	3.99	1.13	1.03
I16	3.94	4.09	1.04	0.98
I17	4.05	4.14	1.10	1.05
I18	4.32	4.38	0.84	0.91
I19	3.82	4.03	1.21	1.12
I20	4.17	4.28	0.95	0.94
I21	4.30	4.45	0.91	0.84
I22	3.40	3.56	1.20	1.20
I23	4.07	4.15	0.96	0.99

### Missing Data

Only one FBS item on the survey had missing data, and that question had only a single missing value. Any responses of “not sure” or “refused” were treated as missing data. Table 6 shows the percentages of respondents who responded with “not sure” or “refused,” and hence treated as missing item data, for those reporting their attachment to cats and dogs. The mean percentage of missing item data for those reporting attachment to cats was 1.7% (SD = 0.82), and those reporting attachment to dogs was 1.1% (SD = 0.66). There were no missing data on gender identity or race. There was 1.2% missing data on the region where respondents lived (urban, suburban, rural), 1% missing on what type of dwelling respondents lived in, and 0.4% missing on marital status. There was 0.5% missing data for education, 3.7% on income, and 2.4% on ethnicity. In data analyses, missing data were handled using Full Information Maximum Likelihood (FIML) in Mplus.

**Table 6:** Missing data for items.

**Item**	**% missing data for cats**	**% missing data for dogs**
I1	0.5	2.1
I2	1.3	0.7
I3	2.8	1.4
I4	1.0	0.7
I5	2.3	2.3
I6	1.8	1.4
I7	2.0	2.1
I8	1.3	0.9
I9	4.1	2.3
I10	2.0	0.2
I11	1.0	0.2
I12	0.5	0.5
I13	1.0	0.9
I14	2.3	0.5
I15	1.5	0.5
I16	1.3	0.7
I17	1.8	1.2
I18	1.3	0.5
I19	1.5	0.9
I20	1.5	0.9
I21	2.3	1.4
I22	2.8	1.9
I23	1.3	1.2

### Reliability

The coefficient (Chronbach's) alpha estimate of reliability of FBS scores for the full sample was 0.962, with a standard error of measurement (SEM) of, +/- 3.23. The coefficient alpha reliability of FBS scores for dog owners was, 0.96, with an estimated SEM of, +/- 3.18. The coefficient alpha estimate of reliability of scale scores for cat owners was, 0.95, with an estimated SEM, +/- 3.37. The differences between these reliability coefficients for dog and cat owners (0.01) and SEMs (0.19) are minor and of no practical significance, and these findings consistent with measurement equivalence.

### Item Analysis

The corrected item-total correlations for items reporting family bondedness to cats and to dogs are shown in Table 7. The results of a test of the equivalence of the distributions of the corrected item-total correlations for cats and dogs was statistically non-significant, Mann-Whitney U = 322.50, standardized test statistic = 1.27, *p* > 0.20. The median corrected item-correlation for cats was 0.73, and for dogs 0.76. The results of a test of equality of medians between the corrected item-total correlations were also statistically non-significant, test statistic (1 df) = 2.17, *p* > 0.10, Yates continuity correction, χ^2^(1) = 1.39, *p* > 0.20. An analysis of variance test of equality of means was also statistically non-significant, F_(1, 44)_ = 1.24, *p* > 0.25. The mean corrected item total correlation for cats was 0.71, and 0.73 for dogs. Levine's test for equal variances of corrected item-total correlations was statistically non-significant, *p* > 0.50. These results were consistent with measurement equivalence.

**Table 7:** Corrected item-total correlations for cat and dog owner item responses.

**Item**	**Cat (*n* = 391)**	**Dog (*n* = 428)**
I1	0.710	0.785
I2	0.817	0.756
I3	0.762	0.772
I4	0.599	0.651
I5	0.751	0.730
I6	0.744	0.817
I7	0.596	0.655
I8	0.683	0.593
I9	0.589	0.675
I10	0.757	0.803
I11	0.756	0.797
I12	0.741	0.750
I13	0.596	0.582
I14	0.746	0.731
I15	0.713	0.666
I16	0.703	0.781
I17	0.765	0.761
I18	0.805	0.801
I19	0.731	0.750
I20	0.764	0.803
I21	0.658	0.788
I22	0.629	0.627
I23	0.691	0.759

### MGCFA-MIMIC Analysis Results

The overall model Chi-square for the full invariance MGCFA-MIMIC model was, $\chi_{\left(1249\right)}^{2}$ = 1568.1, *p* < 0.001. The fit indices were, RMSEA = 0.025, 90% CI: 0.021 - 0.029; and CFI = 0.99; TLI = 0.99. The narrow 90% CI for the RMSEA suggested a reasonably accurate estimate of this fit index (56). These results were consistent with a close-fitting model (50, 56). The results of Chi-square tests were consistent with metric invariance, χ^2^(23) = 20.90, *p* > 0.50, and a similarly statistically non-significant test for invariance of thresholds was consistent with threshold equivalence. The factor loadings, shown in Table 8, were all statistically significant and ranged in value from 0.75 to 1.78. The mean factor loading was 1.22 (SD = 0.30). The R^2^ values for the proportion of item score variance accounted for by the family bondedness latent construct for owners of cats ranged from about 38 to 0.78 (mean = 0.60, SD = 0.12), and for dog owners 0.36 to 0.74 (mean = 0.59, SD = 0.11). There was no evidence suggesting any differential item functioning (DIF) between cat owners and dog owners.

**Table 8:** Raw score factor loadings and R^2^ estimates for item scores.

**Item**	**Factor loading**	**SE**	**z**	***p*-value**	**R^**2**^ cat R^**2**^ dog**
I1	1.78	0.196	9.10	<0.001	0.78 0.64
I2	1.31	0.108	12.14	<0.001	0.65 0.74
I3	1.22	0.087	14.08	<0.001	0.62 0.59
I4	0.94	0.072	13.01	<0.001	0.49 0.44
I5	1.10	0.076	14.50	<0.001	0.57 0.64
I6	1.50	0.131	11.44	<0.001	0.71 0.71
I7	1.00	0.076	13.20	<0.001	0.52 0.44
I8	0.88	0.067	13.10	<0.001	0.46 0.52
I9	0.87	0.061	14.34	<0.001	0.45 0.44
I10	1.70	0.140	12.11	<0.001	0.76 0.73
I11	1.69	0.116	14.55	<0.001	0.76 0.68
I12	1.27	0.091	13.91	<0.001	0.64 0.65
I13	0.80	0.064	12.45	<0.001	0.41 0.44
I14	1.21	0.094	12.92	<0.001	0.61 0.70
I15	0.84	0.060	14.03	<0.001	0.43 0.54
I16	1.30	0.087	15.02	<0.001	0.65 0.60
I17	1.37	0.095	14.34	<0.001	0.67 0.65
I18	1.51	0.127	11.91	<0.001	0.71 0.71
I19	1.11	0.078	14.17	<0.001	0.57 0.62
I20	1.41	0.103	13.78	<0.001	0.68 0.69
I21	1.25	0.098	12.72	<0.001	0.63 0.53
I22	0.75	0.059	12.83	<0.001	0.38 0.36
I23	1.22	0.087	14.06	<0.001	0.62 0.55

The most discriminating items, in the sense of largest factor loadings, were:

- I1 [I love (pet's name)], factor loading (FL) = 1.78;
- I6 [(Pet's name) brings happiness to my life], FL = 1.50;
- I10 [(Pet's name) being in my family makes me happier], FL = 1.70;
- I11 [(Pet's name) makes my family feel complete], FL = 1.69;
- I17 [I tell others that (pet's name) is a member of my family], FL = 1.37;
- I18 [Being with (pet's name) makes me happier], FL = 1.51; and
- I20 [I feel emotionally close to (pet's name)], FL = 1.41.

The content of these items focuses on both degree of bonding with the companion animal, via love and emotional closeness, happiness, and with the feeling the pet/companion animal is a member of the family in the same way as other human family members. These results are consistent with content validity of the items indicative of family bondedness.

A test of the equivalence of the latent variable regression of the independent variables on the family bondedness latent construct across cat and dog owners was statistically non-significant, $\chi_{\left(15\right)}^{2}$ = 12.85, *p* > 0.60, results consistent with invariance of the latent variable regression model between cat and dog owners. The latent variable regression analysis, the results of which are shown in Table 9, suggested males were slightly less bonded with their pets than females, b = −0.24, z = −2.99, *p* < 0.005. Persons living in urban areas were more bonded with their pets than those living in rural areas, b = 0.51, z = 4.47, *p* < 0.001; those living in suburban areas were more bonded than those living in rural areas, b = 0.29, z = 2.88, *p* < 0.005; and those living in urban areas were more bonded than those living in suburban areas, b = 0.23, z = 2.52, *p* < 0.05. Single persons were more bonded with their pets than married persons, b = 0.28, z = 2.81, *p* < 0.01. The estimated R^2^ for the latent construct for cats was 0.08, z = 3.66, *p* < 0.001, and for dogs was 0.06, z = 3.62, *p* < 0.001. Education level, income, and type of housing were found to be unrelated to the degree of family bondedness.

**Table 9:** Results for latent variable regression, HAB the dependent variable.

**IV**	**B**	**SE**	**z**	***p*-value**
Gender	−0.24	0.079	−2.99	<0.005
Education	−0.01	0.041	−0.33	>0.05
Income	<0.001	0.020	0.012	>0.05
Urban vs. rural	0.51	0.114	4.47	<0.001
Suburban vs. rural	0.29	0.100	2.88	<0.005
Urban vs. suburban	0.23	0.093	2.52	<0.02
Apartment vs. house	0.14	0.122	1.17	>0.05
Condo vs. house	0.29	0.191	1.51	>0.05
Duplex vs. house	−0.07	0.252	−0.275	>0.05
Mobile home vs. house	0.30	0.216	1.38	>0.05
Living with partner vs. married	−0.03	0.156	−0.16	>0.05
Divorced vs. married	0.02	0.152	0.11	>0.05
Separated vs. married	−0.005	0.313	−0.02	>0.05
Widowed vs. married	−0.31	0.270	−1.13	>0.05
Single vs. married	0.28	0.099	2.81	<0.01
Hispanic vs. non-Hispanic	0.02	0.098	0.20	>0.05

Results suggested there was no statistically significant difference between family bondedness of Hispanic vs. non-Hispanic pet owners, b = 0.02, z = 0.20, *p* > 0.50. However, these findings should be taken as tentative given the small sample size of Hispanic respondents, *n* = 105, 12.8% of the sample (56). Future research needs to address the measurement equivalence of FBS scores across Hispanic and non-Hispanic populations as well as further investigation of the degree of bondedness between Hispanic and non-Hispanic populations. There were no statistically significant paths indicated from any of the independent variables to FBS items, results consistent with absence of DIF with respect to these independent variables (56).

Overall, these findings were consistent with measurement equivalence of FBS scores for those reporting family bondedness with cats and dogs. The results suggested that for a given value of the family bondedness latent construct, the expected observed scores on the FBS will be the same for those reporting on family bondedness with cats and dogs, controlling for the observed variables in the latent construct regression.

## Discussion and Conclusion

Results were consistent with configural, metric, and scalar invariance; with the absence of differential item functioning as implied by the MGCFA-MIMIC model results; with comparable reliability coefficients and standard errors of measurement; and with comparable corrected item-total correlations for FBS item scores. These results provide multiple forms of evidence for measurement equivalence of person's scores for their family bondedness to cats and dogs (50). The results were also consistent with the FBS being a unidimensional scale. These results, pending further results of validity relevant research, support the use of the FBS in HAI and HAB research by veterinarians, social workers, psychologists, and others investigating the relationships between family bondedness and other relevant variables. These results also suggest the FBS addresses important limitations in HAI and HAB measurement scales discussed by Rodriguez et al. (32), and Branson et al. (47). The addressed limitations include evidence for measurement equivalence across different animal species, specifically cat and dogs; and the need for short-form scales.

A strength of the current study results is that the findings of measurement equivalence controlled for the relationships the independent variables in the latent regression had with the family bondedness latent construct. There are also limitations. These concern the significant differences between sample income levels, racial percentages, and education levels as compared with U.S. population values. These differences raise questions about the generalizability of the results of this study to the broader U.S. population. Future research on FBS scores should entail an emphasis on obtaining more representative samples of respondents with respect to these variables.

The current study is only a first step in building a case for validity of scores on the FBS representing the degree to which a pet owner is emotionally bonded with their pet in a manner equivalent to their emotional bond with other human family members. Much research needs to be done to build a strong case for validity of scores on this scale, as elaborated by Kane (57). Further research on measurement equivalence of FBS scores across different types of companion animals is needed to build a more complete case for use of this scale in research focusing on family bondedness with a range of companion animals and human populations. Measurement equivalence evidence is needed to confirm the results of the current study, as well as evidence for equivalence of measurement between various populations of persons, including equivalence between those self-identifying as male or female, Black and White, as well as other racial groups; and Hispanic and non-Hispanic ethnic groups, among other comparisons. Age-related measurement equivalence studies also need to be done. For those interested in investigating differences between bondedness with unusual companion animals such as birds, snakes, and other exotics, further measurement equivalence studies of the FBS need to be conducted before carrying out such research. If the items on this scale are to be translated into different languages, then measurement equivalence studies of these different forms will need to be conducted.

Further research is also needed to provide different forms of validity evidence (57). Criterion-related validity evidence and convergent/divergent validity evidence, in particular, are needed. Psychometric research on FBS scores using a variety of different measurement theories also needs to be conducted. For example, studies of FBS scores using Item Response Theory need to be conducted. Consistency in results across these studies would help confirm the validity of results of the current study as well as these other investigations.

With further validity evidence, the brevity of this scale, its easy readability level, and the evidence for measurement equivalence would make the FBS useful for research, program evaluations, and other forms of practical application involving HAI and HAB research involving the degree to which pet owners are emotionally bonded to their companion animals as family members. Potential uses of the FBS include its use for any research comparing family bondedness of persons to companion animals that are cats or dogs. It also shows promise for use in outcome research, and for research investigating the possible mediating and/or moderating effect of family bondedness on outcomes of programs and interventions, such as animal-assisted therapy. It also shows promise for use in research on pet parenting styles, in particular on how emotional bonding with the pet may influence pet parenting, and vice versa, how pet parenting styles may impact the emotional bond pet owners have with their pets.

Finally, the results of the current study suggest that, contingent on further validity evidence, the FBS could be used in veterinary practice, as well as practice in social work, psychology, and other relevant disciplines. The FBS could be used as a part of any complete assessment of a veterinary case in which the degree of family bondedness with a pet plays an important role. For example, veterinarian's approach to euthanasia discussions with persons with very high family bondedness may also need to be different than with those with lower family bondedness. Grief work by veterinary social workers with persons whose companion animals have died might need to be different for those with high FBS scores than for those with low FBS scores. If clinical evidence suggests a pet owner with a higher degree of family bondedness with their companion animal might be more likely to faithfully carry out a post-surgery plan of care than an owner with a lower level of family bondedness, then knowledge of FBS scores would be useful in not only formulating the plan of care but also in explaining and persuading the pet's owner to implement the plan. In cases in which a supplementary professional is involved, such as a veterinary social worker or other social service professional, the FBS could be used as a part of a comprehensive psychosocial assessment of the family of which the companion animal is a part.

## Data Availability Statement

The datasets presented in this article are not readily available because the data were approved for access by the two authors only. Requests to access the datasets should be directed to William R. Nugent, wnugent@utk.edu.

## Ethics Statement

The studies involving human participants were reviewed and approved by the University of Tennessee-Knoxville Institutional Review Board. Written informed consent for participation was not required for this study in accordance with the national legislation and the institutional requirements.

## Author Contributions

WN created the Family Bondedness Scale and conducted the data analyses. LD designed and conducted the survey. WN and LD both contributed to the writing of the article. All authors contributed to the article and approved the submitted version.

## Funding

The funding agency was Maddie's Fund. There are funds allocated in this grant for publication fees. The award number A22-0133-00120.

## Conflict of Interest

The authors declare that the research was conducted in the absence of any commercial or financial relationships that could be construed as a potential conflict of interest.

## Publisher's Note

All claims expressed in this article are solely those of the authors and do not necessarily represent those of their affiliated organizations, or those of the publisher, the editors and the reviewers. Any product that may be evaluated in this article, or claim that may be made by its manufacturer, is not guaranteed or endorsed by the publisher.
